# Primary care patients with drug use identified by screening self-medicate with alcohol and other drugs for chronic pain

**DOI:** 10.1186/1940-0640-10-S2-P15

**Published:** 2015-09-24

**Authors:** Richard Saitz, Daniel P Alford, Jacqueline S German, Jeffrey H Samet, Debbie M Cheng, Christine A Lloyd-Travaglini

**Affiliations:** 1 Community Health Sciences and General Internal Medicine, Boston University Schools of Public Health & Medicine/Boston Medical Center, Boston, USA; 2 General Internal Medicine, Boston University School of Medicine/Boston Medical Center, Boston, USA; 3 Community Health Sciences, Boston University Schools of Public Health & Medicine/Boston Medical Center, Boston, USA; 4 Biostatistics and General Internal Medicine, Boston University Schools of Public Health & Medicine/Boston Medical Center, Boston, USA; 5 Data Coordinating Center, Boston University School of Public Health, Boston, USA

## Background

Chronic pain is common among patients with drug use disorders. The prevalence of chronic pain and its consequences in primary care patients who use drugs is unknown and deserves further study.

The objectives are to determine the prevalence of chronic pain and pain-related dysfunction among primary care patients who screen positive for drug use. To examine the prevalence of substance use to self-medicate chronic pain in this population.

## Material and methods

Cross-sectional analysis of adult patients who screened positive for any illicit drug use or prescription drug misuse, recruited from an urban, hospital-based primary care practice. Both pain and pain-related dysfunction were assessed by numeric rating scales, and grouped as: (0) None, (1-3) mild, (4-6) moderate, (7-10) severe. Questions were asked about the use of substances to treat pain.

## Results

Among 589 participants, chronic pain was reported by 87%, with 13% mild, 25% moderate and 50% severe. Pain-related dysfunction was reported by 74% of participants, with 15% mild, 23% moderate, and 36% severe. Among those who used marijuana, cocaine, and/or heroin, 51% (283/576) reported using to treat pain. Of the 121 with prescription drug misuse, 81% used to treat their pain. Of the 265 participants who reported drinking any alcohol, 38% did so to treat pain compared to 61% of the 114 with heavy alcohol use.

## Conclusions

Chronic pain and pain-related dysfunction were the norm for primary care patients who screened positive for drug use. Almost half of these patients reported severe pain and approximately a third reported both severe pain and severe pain-related dysfunction. Many patients using illicit drugs, misusing prescription drugs and using alcohol reported doing so in order to self-medicate their pain. Pain needs to be addressed when patients are counseled about their substance use.

